# Extraction and reconstitution of membrane proteins into lipid nanodiscs encased by zwitterionic styrene-maleic amide copolymers

**DOI:** 10.1038/s41598-020-66852-7

**Published:** 2020-06-18

**Authors:** Mariana C. Fiori, Wan Zheng, Elizabeth Kamilar, Geuel Simiyu, Guillermo A. Altenberg, Hongjun Liang

**Affiliations:** Department of Cell Physiology and Molecular Biophysics, and Center for Membrane Protein Research, Texas Tech University Health Sciences Center, Lubbock, Texas USA

**Keywords:** Membrane structure and assembly, Nanostructures, Nanoscale materials, Polymer chemistry, Biophysical methods

## Abstract

Membrane proteins can be reconstituted in polymer-encased nanodiscs for studies under near-physiological conditions and in the absence of detergents, but traditional styrene-maleic acid copolymers used for this purpose suffer severely from buffer incompatibilities. We have recently introduced zwitterionic styrene-maleic amide copolymers (zSMAs) to overcome this limitation. Here, we compared the extraction and reconstitution of membrane proteins into lipid nanodiscs by a series of zSMAs with different styrene:maleic amide molar ratios, chain sizes, and molecular weight distributions. These copolymers solubilize, stabilize, and support membrane proteins in nanodiscs with different efficiencies depending on both the structure of the copolymers and the membrane proteins.

## Introduction

Understanding membrane proteins at the functional and structural levels is critical in biomedicine because most clinically-used drugs target these proteins^1^. In contrast to soluble proteins, which are stable in water-based solutions, integral membrane proteins need an environment that can accommodate hydrophobic and hydrophilic moieties to interact with the membrane lipid bilayer and the aqueous solutions bathing the membrane, respectively. Membrane proteins can be extracted from phospholipid membranes with detergents, and they can be purified in their detergent-solubilized forms. Then, they can be analyzed while solubilized in detergent micelles or other amphipathic molecules, or reconstituted into lipid-bilayer membranes. The use of detergents is widespread because of its simplicity and the availability of a variety of detergents with different properties. However, detergent micelles are not ideal membrane protein-supporting platforms. The dynamic equilibrium between detergent monomers and micelles, and differences in physicochemical properties between detergent micelles and biomembranes (*e.g*., curvature, lateral pressure profile, thickness) are some of the causes of the decrease in membrane-protein stability in detergents^2–5^. In addition, the structure and function of membrane proteins can be affected when the proteins are in detergent micelles^6–11^, which is leading to the increased use of biomembrane-based platforms.

Lipid nanodiscs (LNDs) are one of the frequently used platforms for reconstitution of membrane proteins in biomembranes. LNDs are formed by a small lipid-bilayer patch surrounded by two copies of belt-like membrane scaffold proteins that are derived from apolipoprotein A1^11^. Membrane proteins in nanodiscs are accessible from both sides of the membrane, and part of their appeal is that they can exist as monodisperse proteolipid nanostructures that can be studied in the same way as soluble proteins^11^. As such, nanodiscs can be used in combination with varied methodologies that include NMR and single-particle cryo-electron microscopy^10–13^.

Styrene-maleic acid (SMA)-lipid particles (SMALPs) are a variant of LNDs where amphipathic SMA copolymers substitute the membrane scaffold proteins^14–16^. Two features of SMAs make them highly desirable: 1) SMALPs can be produced by direct membrane solubilization with SMA copolymers, maintaining the membrane proteins in a lipid bilayer and avoiding the use of detergents^14–16^, and 2) SMALPs can also be formed from proteoliposomes of known lipid composition, which is useful to study the role of membrane lipids in determining membrane protein structure and function^14–16^. The use of SMAs has increased significantly during the last few years, but incompatibility with common buffers is a significant problem. These incompatibilities include solutions of pH ≤ 6 and the presence of millimolar concentrations of Ca^2+^, Mg^2+^ and polyvalent cations in general^14,17,18^. Precipitation of SMA and SMALPs occurs because of hydrophobic interactions when it becomes neutral at low pH or as a result of association of the polyvalent cations with the negative charges of the carboxyl acids. These incompatibilities preclude experiments that have to be performed at pH ≤ 6 (*e.g*., studies of H^+^ pumps and H^+^-gated channels), require Mg^2+^ or other divalent cations (*e.g*., most ATPases), or are regulated by Ca^2+^ (*e.g*., closure of connexin hemichannels). Also, SMA carboxyl groups can interfere with standard His tag-based purification of membrane proteins through coordination of the carboxyl acids of SMA with transition metal ions used in immobilized metal affinity chromatography, or shielding of the affinity purification tag^17,18^. We have solved some of the problems associated with the use of SMAs by designing and synthesizing new styrene-maleic amide copolymers where the maleic acid moieties of traditional SMA were replaced with maleic amide moieties conjugated to zwitterionic phosphatidylcholine (PC) groups^19^. Even though the new copolymers do not contain maleic acid, we decided to refer to them as zSMAs ("z" for zwitterionic). The main reasons for this are the broad use of SMA and SMALP, and the fact that “MA” can be still used to represent the new maleic amide moieties. We showed that these new copolymers are able to solubilize membrane proteins into nanodiscs of controlled sizes^19^. In our previous study, we focused on 1:1 styrene:maleic amide molar ratio (St:MA) zSMAs with molecular weight greater than 10 kDa^19^. In reference to studies on traditional SMAs that solubilize membranes into nanodiscs, oftentimes the commercially-available SMAs with molecular weight smaller than 10 kDa were used^20–28^, and the best membrane solubilization was reported with 2:1 St:MA SMAs^23,27,29^. Here, we compared the extraction/reconstitution of membrane proteins into LNDs by a series of zSMAs with different St:MAs (*i.e*., 1:1, 2:1, and 3:1), molecular weight, and polydispersity indices (*i.e*., in-house prepared zSMAs *via* controlled/“living” polymerization *vs* zSMAs derived from commercial SMAs). Our results show that the zSMAs solubilize, stabilize, and support membrane proteins in nanodiscs with different efficiency depending on both the structure of the copolymers and the type of the membrane proteins.

## Results and Discussion

### zSMA copolymers

**Figure 1:**
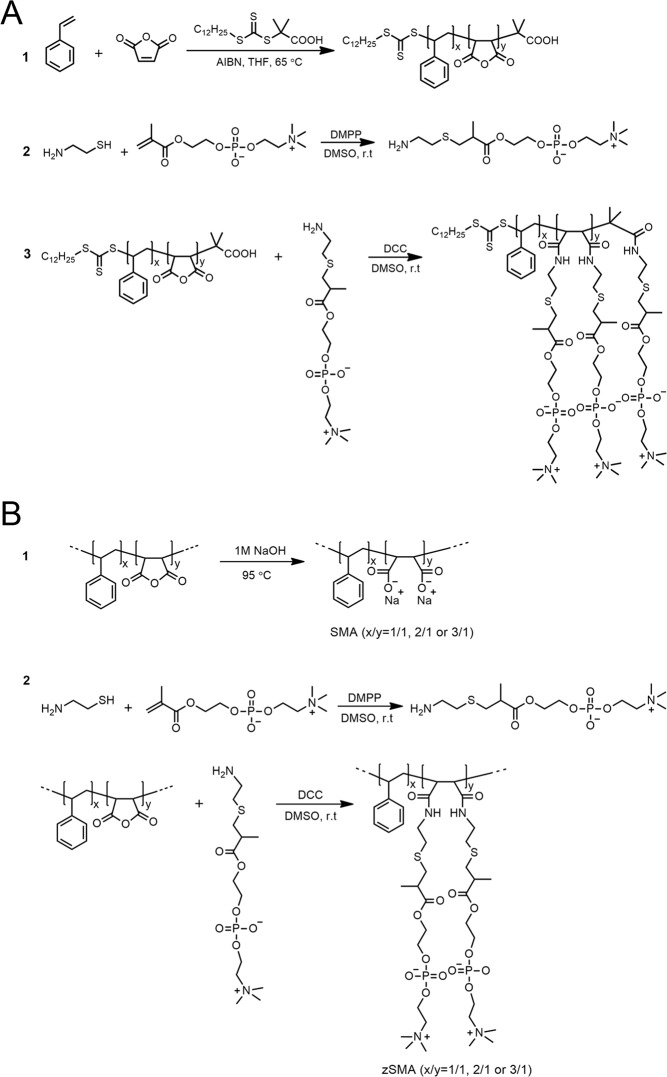
Reaction designs used to prepare zSMAs *via* RAFT polymerization (**A**) and SMAs and zSMAs using Lipodisq P[St-*ran*-MA] copolymers as precursors (**B**).

We compared five different zSMAs in this study. Two of the zSMAs with well-defined sizes and different St:MAs (1:1 and 2:1) were derived from styrene-maleic anhydride random copolymers (P(St-*ran*-MA)) produced by reversible addition-fragmentation chain-transfer (RAFT) polymerization (Fig. 1A). For convenience, we simply refer to these copolymers as zSMAs. It should be noted that for the synthesis of P(St-*ran*-MA) with initial St:MA feeding ratios >1, there is a possibility of a gradient increase of styrene repeating units that could lead to a polystyrene homopolymer segment at the end of individual P(St-*ran*-MA) chains. To minimize this possibility, we controlled the conversion of styrene in the P(St-*ran*-MA) to be ~20%. We also prepared three different chain sizes of the 2:1 zSMA to test the effect of molecular weight on membrane solubilization. Three additional zwitterionic copolymers were synthesized using Malvern Lipodisq P[St-*ran*-MA] copolymers as precursors (Fig. 1B). These are referred to as 1:1, 2:1 and 3:1 M zSMAs (Fig. 1B). As controls, we also prepared conventional SMA copolymers using the same Lipodisq copolymers as precursors (Fig. 1B).

### Structural characterization of the copolymers

The molecular weights and polydispersity indices (PDIs) of the different P(St-*ran*-MA) copolymers are presented in Table 1. As expected, the copolymers prepared *via* RAFT polymerization have much smaller PDIs than those received from Malvern, indicating less variation in their chain size distribution (Supplementary Fig. 1A *vs* 1B). Since all the Lipodisq P(St-*ran*-MA) copolymers have similar sizes (~5–6 kDa), we designed the RAFT P(St-*ran*-MA) copolymers to have a similar size (~6–7 kDa). For the 2:1 RAFT P(St-*ran*-MA), we also prepared two other sizes at ~2x and 0.5x of the Malvern copolymer size to examine the effect of chain size on membrane solubilization (Table 1). We used proton nuclear magnetic resonance spectroscopy (^1^H NMR) to confirm the successful synthesis of each reaction intermediates, as well as to determine the chain size and St:MA of each copolymer product (Table 1). Examples of ^1^H NMR spectra are presented in Supplementary Fig. 2A,B. We also adapted an alternative method based on UV spectroscopy (Supplementary Fig. 3) to validate the St:MA ratios calculated from the ^1^H NMR analysis. In tetrahydrofuran (THF), the styrene moiety has a characteristic UV absorption peak at 245 nm that does not overlap with any other absorption features of the copolymers. We measured the molar absorption coefficient (ɛ_245_) of our well-defined P(St-*ran*-MA) copolymers (Supplementary Fig. 3B and Table 1), and compared the values to the expected ɛ_245_ of these copolymers calculated on the basis of the St:MA ratios derived from the ^1^H NMR analysis using a standard polystyrene sample as reference (PSt_62_, Supplementary Fig. 3A). We found that the measured ɛ_245_ of P(St-*ran*-MA) copolymers agreed nicely (Supplementary Fig. 3C) with those expected values when the molar fractions of styrene repeating units in these polymers changed from 100% in PSt, to 67% (in P(St-*ran*-MA) copolymers polymers with St:MA~2:1 as determined by ^1^H NMR), and to 50% (in P(St-*ran*-MA copolymers with St:MA~1:1 as determined by ^1^H NMR). Note that we did not measure the ɛ_245_ of Malvern Lipodisq P(St-*ran*-MA) copolymers because of their polydisperse nature.

**Table 1:** Structural characterization data of P(St-*ran*-MA) copolymers.

Sample Name	St:MA^c^	M_n_ (kDa)^c^	M_n_ (kDa)^d^	PDI^d^	ε_245_^e^
P(St_31_-*ran*-MA_31_)^a^	1.00/1.00	6.6	6.7	1.05	12.8
P(St_18_-*ran*-MA_9_)^a^	2.00/1.00	3.1	3.1	1.05	17.2
P(St_40_-*ran*-MA_21_)^a^	1.93/1.00	6.6	6.4	1.04	17.7
P(St_76_-*ran*-MA_39_)^a^	1.95/1.00	12.6	12.0	1.13	18.0
P(St-*ran*-MA)^b^	0.95/1.00	/	4.6	1.22	/
P(St-*ran*-MA)^b^	2.00/1.00	/	5.0	1.52	/
P(St-*ran*-MA)^b^	2.86/1.00	/	5.7	1.59	/

^a^Synthesized *via* RAFT polymerization; ^b^Lipodisq copolymers obtained from Malvern Cosmeceutics; ^c^Obtained by NMR analysis; ^d^Obtained by GPC analysis; ^e^The ε_245_ of polystyrene (PSt_62_) was 25.6 mM^−1^ cm^−1^ (see Supplementary Fig. 2).

### Solubilization of HR and MsbA in crude membranes by zSMAs

For the solubilization test we used two very different membrane proteins: *Natronomonas pharaonis* halorhodopsin (HR) and *Salmonella typhimurium* MsbA. HR is a photosensitive Cl^−^ pump that belongs to the 7-transmembrane receptor protein family, and MsbA is a twelve-transmembrane helix homodimer that belongs to ATP-binding cassette (ABC) superfamily^6,30^. HR uses the energy of photons absorbed by all-trans retinal for Cl^−^ influx, whereas MsbA hydrolyzes ATP and uses the resulting energy for translocation of lipid A from the inner leaflet to the outer leaflet of the membrane^30–32^. A main goal of this study was to identify how variations in the incubation conditions affect solubilization of the recombinant proteins from crude membranes by different zSMA copolymers. For these studies, the total protein concentration of the crude membranes was kept at <3 mg/mL. Recombinant membrane proteins are most frequently expressed in mammalian or insect cells, yeast (*P. pastoris* or *S. cerevisiae*) or a variety of *E. coli* strains, including BL21 cells^33,34^. The characteristics of the cell membranes used in different expression systems vary significantly, which affects solubilization^20,35–37^. Even though using the same expression system for HR and MsbA does not assure the same membrane composition, we expressed HR and MsbA in the same cells (BL21 *E. coli*) to minimize differences and simplify comparison of HR and MsbA solubilization, although lipid-to-protein ratios are not exactly the same for both proteins since HR expresses at higher levels than MsbA.

With these considerations in mind, we explored the effects of several factors that can affect solubilization: temperature, incubation time, salt concentration, pH, St:MA, and copolymer concentration and size. Increasing copolymer concentration from 1% to 2.5% had no effect or decreased solubilization (Supplementary Fig. 4A). In additional experiments, increasing the concentration of zSMAs and M zSMAs to 5% decreased solubilization by 26 ± 5% (P < 0.001 *vs* 1%; n = 8). Based on these observations, we conclude that under the conditions of our experiments increasing copolymer concentration beyond 1% does not improve zSMAs solubilization efficiency. Since increasing the temperature of incubation from room temperature (RT) to 37 °C had small detrimental or no effects on HR solubilization (Supplementary Fig. 4B), we settled for solubilization of HR at RT. As for MsbA, we decided to use 37 °C because this temperature is well tolerated by MsbA, and increased temperature was reported to improve solubilization by traditional SMA copolymers^26,37^. Data supporting the use of 2-h MsbA solubilization at temperatures higher than 4° are presented in Supplementary Fig. 5. In any case, increasing the temperature from RT to 37 °C has only minor effects on solubilization, and therefore solubilization by zSMA and M zSMA copolymers can be carried out at RT or above without compromising efficiency. Increasing ionic strength is believed to improve solubilization by traditional SMAs due to a decrease of the electrostatic repulsion between the negatively charged membranes and maleic acid groups, which facilitates SMA binding, the first step in membrane solubilization^26,27,29^. Generally, the presence of 100–200 mM NaCl is sufficient, and high salt concentration can be detrimental because the negative charges on traditional SMAs are important to keep the copolymers soluble in aqueous solution^27^. These effects should not be as important for the zSMAs. In fact, we found only minor differences between solubilization in the presence of 150 or 500 mM NaCl for the zwitterionic copolymers (Supplementary Fig. 6A). Since there were small improvements with 500 mM *vs* 150 mM NaCl for the 1:1 zSMA and M zSMA (HR), and no effects for the other copolymers in the HR experiments or MsbA, we decided to use the 500 mM NaCl, but either concentration should work equally well. We also explored the effects of solution pH on the copolymers solubilization efficiency, testing pH 6.5, 7.5 and 8.3. We observed a decrease in solubilization by 2:1 SMA at the more acid pH (Supplementary Fig. 6B), which is not surprising given the tendency of traditional SMA to precipitate at low pH^14,17–19^. There were significant increases in HR solubilization by 1:1 M zSMA at pH 8.3 *vs* 6.5 (P < 0.005) and 2:1 M zSMA at pH 7.5 *vs* 6.5 (P < 0.01), and in MsbA solubilization by 1:1 zSMA at pH 7.5 *vs* 6.5 (P < 0.01). Since there were no statistical differences between pH 7.5 and 8.3, we settled for the use of pH 7.5, but any pH ≥7 should work well for solubilization by zSMAs and M zSMAs (Supplementary Fig. 6B). Based on the effects of copolymer concentration, incubation temperature and time, as well as salt concentration and pH, our basic solubilization conditions consisted of 1% copolymer, 500 mM NaCl and pH 7.5, with incubation for 2 h at RT temperature for HR and 37 °C for MsbA. However, our comparative studies also suggest that solubilization of membrane proteins by zSMAs can be accomplished under a broad set of conditions without compromising the efficiency significantly.

**Figure 2:**
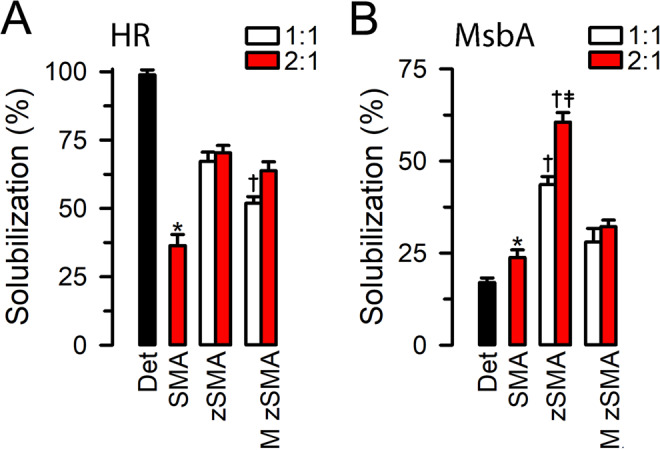
Solubilization of recombinant proteins from *E. coli* crude membranes. Membranes were incubated in 500 mM NaCl and 50 mM Tris/HCl, with 10% glycerol, pH 7.5, and 1% (w/v) of copolymer, and the samples were incubated for 2 h at RT for HR or at 37 °C for MsbA. For the detergent experiments, DDM was used at 1.5% for HR and a mixture of 2% DDM and 0.04% sodium cholate was used for MsbA. (**A**) Solubilization of HR. Det (n = 21): detergent; SMA (n = 20): 2:1 SMA from Malvern; zSMA: our synthetic zwitterionic copolymers that include 1:1 zSMA (n = 21) and 2:1 zSMA (n = 26) derived from the RAFT P(St-*ran*-MA) with molecular weights of 6.7 and 6.4 kDa, respectively; M zSMA: zwitterionic copolymers synthesized from Malvern SMA precursors that include 1:1 M zSMA (n = 15) and 2:1 M zSMA (n = 23) derived from the Malvern P(St-*ran*-MA) with molecular weights of 4.6 and 5.0 kDa, respectively. The white and red colors indicate 1:1 and 2:1 St:MA, respectively. Det solubilization was significantly higher than that with all polymers (P < 0.001). *Denotes P < 0.001 *vs* other copolymers; ^†^ denotes P < 0.02 *vs* 2:1 zSMA and 2:1 M zSMA. (**B**) Solubilization of MsbA. Det: n = 21; SMA: n = 20; 1:1 zSMA: n = 21; 2:1 zSMA: n = 25; 1:1 M zSMA: n = 15; and 2:1 M zSMA: n = 23. Det solubilization was significantly lower than that with all polymers (P < 0.001). *Denotes P < 0.01 *vs* other copolymers, except for 1:1 M zSMA (NS); ^†^Denotes P < 0.001 *vs* corresponding M zSMAs; ^‡^Denotes P<0.001 *vs* 1:1 zSMA. Data are means ± SEM.

Figure 2 shows the efficiency of solubilization of HR and MsbA under our basic solubilization conditions. Individual experimental results obtained over the course of ~2 years are shown in Supplementary Fig. 7. For HR (Fig. 2A), which is extracted very well from crude membranes by detergent (1.5% *n*-dodecyl-β-D-maltopyranoside; DDM), the solubilization by copolymers was less efficient, but still >50% for the zSMA and M zSMA copolymers in contrast to <40% for 2:1 SMA. Taken together, both sets of zwitterionic copolymers with different St:MAs displayed a good extraction efficiency for HR. For MsbA (Fig. 2B), which is not extracted very efficiently by detergent (2% DDM/0.04% sodium cholate), the copolymers did better than the detergent, in particular the zSMA copolymers. Of these, the 2:1 zSMA showed the best performance, with solubilization of ~60% of the MsbA in crude membranes, ~3.5-folds the extraction efficiency by the detergent mix. An increase in solubilization efficiency by SMAs with St:MA molar ratios 2:1 *vs* 1:1 has been reported^23,27,29^, and our data suggest that this improved efficiency depends, at least in part, on the target protein (Fig. 2). We also noticed that zSMAs outperformed M zSMAs by ~2-folds under otherwise similar conditions. This significant difference for MsbA solubilization between zSMAs and M zSMAs could result from their different microstructures and polydisperse indices (Supplementary Fig. 1 and Table 1) due to the different synthetic routes, as previous studies suggested that molecular weight distribution could play a role in the solubilization/reconstitution^21,25^. However, we are not clear why HR extraction is less sensitive to this difference (Fig. 2A) than MsbA, and we cannot rule out other possibilities. A relatively long styrene-rich hydrophobic tail of SMA copolymers at St:MAs higher than 1 has been associated with reduced solubilization efficiency^25,28,38^. However, HR and MsbA solubilization efficiencies by 2:1 zSMA were very similar (60–65%), and better than solubilization by 2:1 SMA tested under the same conditions for both proteins (Fig. 2), and comparable to those for extraction of a variety of proteins from membranes by SMAs^23,39^. This suggests that the early termination of the polymerization reaction in our synthesis helps minimize the potential styrene-rich hydrophobic tail and its associated adverse effect on solubilization. Overall, these results showed that our zSMA and M zSMA copolymers are practical alternatives to detergents for the solubilization of membrane proteins, have the distinct advantage of the direct reconstitution in a lipid bilayer membrane, and avoid the precipitation that occurs with the use of SMAs at pH ≤ 6 or in the presence of polycations^19^.

**Figure 3:**
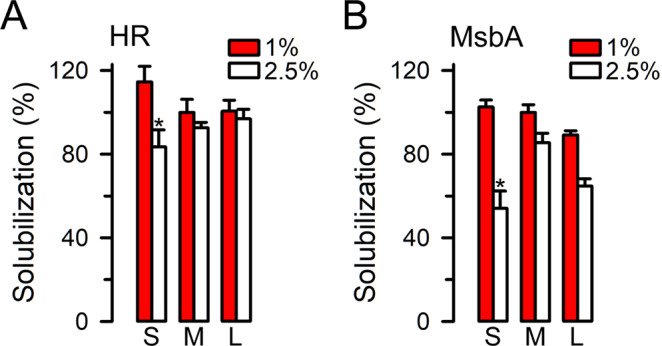
Effects of copolymer size on HR and MsbA solubilization. Solubilization conditions were those of the basic protocol, except that 1% or 2.5% of the 2:1 zSMA with three different chain sizes were used: the S, M, and L represent zSMA derived from RAFT P(St-*ran*-MA) with molecular weights of 3.1, 6.4 and 12.0 kDa, respectively. Data were normalized to the average of 1% zSMA with medium molecular weight (“M”). *Denotes P < 0.05 *vs* the corresponding 1% value. Data are means ± SEM (n = 3 *per* condition).

Figure 3 shows the effects of 2:1 zSMA copolymers of different molecular weights on solubilization, and a representative Western blot is shown in Supplementary Fig. 8. The solubilization conditions were those of the basic protocol used for the experiments in Fig. 2. Here we tested small-, medium-, and large-size zSMAs derived from RAFT P(St-*ran*-MA) copolymers with molecular weights of 3.1, 6.4 and 12.0 kDa, respectively. The solubilization by the small-size zSMA was reduced at 2.5 *vs* 1% copolymer concentration for both HR (P < 0.05) and MsbA (P < 0.01), whereas no differences were detected for the solubilization between the three copolymers at the preferred concentration of 1%. These results suggest that zSMA copolymers perform essentially similarly in the molecular weight range tested, and P(St-*ran*-MA) copolymers in the 3- to 12-kDa size range can be safely used as a starting point for further development of zSMAs.

**Figure 4:**
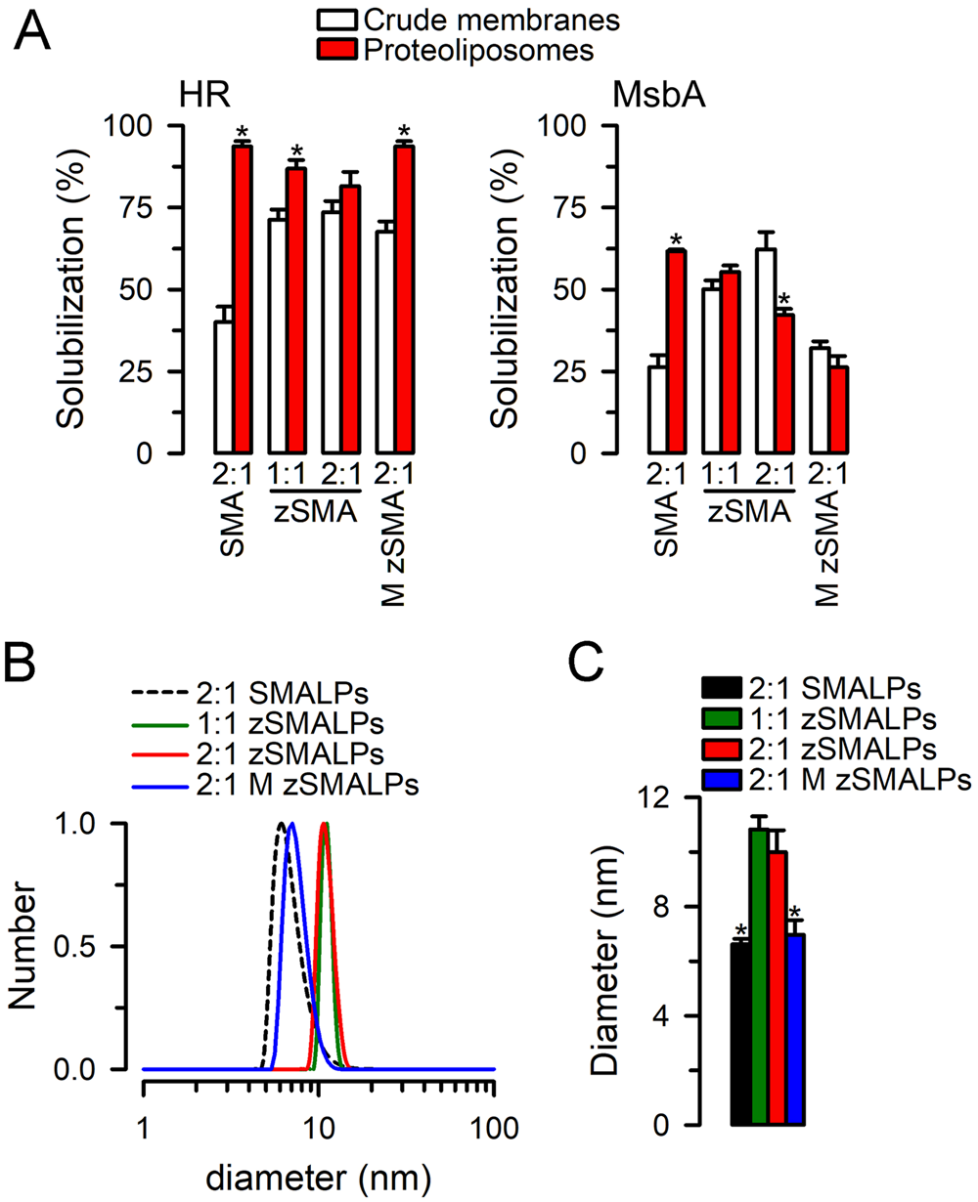
Solubilization of reconstituted HR and MsbA in liposomes. (**A**) HR and MsbA solubilization. HR and MsbA in crude *E. coli* membranes or purified HR and MsbA reconstituted in liposomes formed by *E. coli* lipids were solubilized under the conditions of the basic protocol (Fig. 2). The 1:1 and 2:1 zSMA were derived from the RAFT P(St-*ran*-MA) copolymers with molecular weights of 6.7 and 6.4 kDa, respectively, and the M zSMA was derived from the 2:1 Malvern P(St-*ran*-MA) copolymers with molecular weight of 5.0 kDa. *Denotes P < 0.05 *vs* the corresponding value for crude membranes. Data are means ± SEM (n = 4–7 *per* condition). (**B**) Typical examples illustrating the hydrodynamic diameter distributions of zSMALPs and M zSMALPs determined by dynamic light scattering. The 2:1 SMALPs were prepared using the SMA derived from the 2:1 Malvern P(St-*ran*-MA) copolymers with molecular weight of 5.0 kDa, and the zSMALPs and M zSMALPs were prepared using corresponding zSMAs and M zSMA as described in A. (**C**) Summary of the average hydrodynamic diameter of nanodiscs formed by solubilization of proteoliposomes containing purified HR or MsbA. Values for HR- and MsbA-loaded nanodiscs were not statistically different and were pooled. SMALPs (n = 5), 1:1 zSMALPs (n = 9), 2:1 zSMALPs (n = 9) and 2:1 M zSMALPs (n = 8). Data are means ± SEM; *Denotes P < 0.01 *vs* zSMALPs.

As mentioned in the Introduction, one of the uses of SMAs and zSMAs is in the solubilization of purified membrane proteins reconstituted in liposomes of defined composition. Such experiments can be useful to determine the effects of the bilayer composition on membrane proteins. In spite of the versatility of zSMAs and M zSMAs as solubilization agents, our data in membranes with similar lipid composition and under the same experimental conditions show significant differences in the solubilization of MsbA from BL21 *E. coli* membranes and liposomes formed by *E. coli* polar lipids. This is consistent with previous reports showing that the type and state of the membrane affects solubilization by the copolymers^20,35–37^. In general, solubilization of HR from proteoliposomes was better, and the same was true for MsbA solubilization by 2:1 SMA, with the differences being more marked for the latter (Fig. 4A). In contrast, MsbA solubilization by 2:1 zSMA from crude membranes was more efficient than from proteoliposomes (Fig. 4A). The results do not support a uniform role of the protein:lipid ratios between the membranes and proteoliposomes (higher in the membranes) that explains the observed differences in solubilization, since the membrane protein and polymer are clearly significant factors. More studies will be needed to explain the differences presented in Fig. 4A. Nevertheless, the differences in the efficiency of MsbA extraction from crude membranes and proteoliposomes by the zSMAs were not major, and the results show that solubilization of both membrane proteins from proteoliposomes by zSMAs and M zSMAs is straightforward and feasible (Fig. 4A).

Figure 4B shows size distributions of the copolymer nanodiscs formed by solubilization of HR or MsbA reconstituted in proteoliposomes. As shown in Fig. 4B,C, the 1:1 and 2:1 zSMALPs have a very focused size distribution with an average diameter of 10–11 nm. In contrast, the 2:1 SMALPs and M zSMALPs, both of which are encased by copolymers derived from Malvern Lipodisq P[St-*ran*-MA] precursors, have a relatively broader size distribution with a peak diameter of ~7 nm. Identical DLS analysis yielded diameters of 6.0 ± 0.2 nm for detergent-solubilized MsbA (n = 21) and 9.7 ± 0.3 nm for MsbA in traditional lipid nanodiscs (n = 5). Although we do not have a definitive explanation for the differences in apparent sizes, the similarity between the diameters calculated from DLS for MsbA in traditional LNDs and zSMALPs suggests that the latter form similar nanodisc-like structures, whereas the MsbA-loaded SMALPs and M zSMALPs may consist of a mixture of nanodiscs and smaller polymer-lipid nanoparticles due to the polydisperse nature of the commercial P[St-*ran*-MA] precursors. Overall, our results are consistent with the formation of nanodisc-size structures following solubilization by 2:1 SMA, zSMAs and M zSMAs.

**Figure 5:**
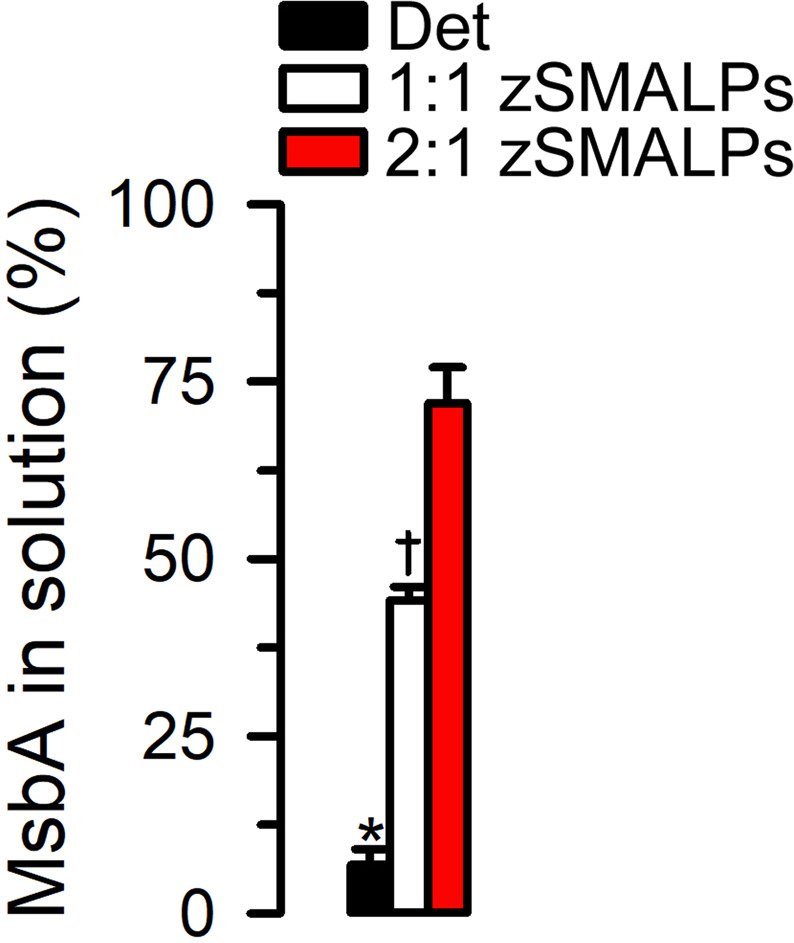
Thermal stability of solubilized MsbA. Purified MsbA was studied in detergent (Det) and zSMALPs (solubilized from proteoliposomes). For these experiments the samples were heated to 65 °C for 15 min and MsbA in the supernatant, after centrifugation at 100,000 g for 45 min, was quantified on Western blots probed with an anti-His antibody. The 1:1 and 2:1 zSMALPs were prepared using the corresponding zSMAs derived from RAFT P(St-*ran*-MA) copolymers with molecular weights of 6.7 and 6.4 kDa, respectively. *Denotes P < 0.001 *vs* both copolymers; ^†^Denotes P < 0.002 vs 2:1 zSMA. Data are means ± SEM (n = 4 *per* condition).

One potential advantage of the reconstitution in zSMALPs *vs* solubilization in detergent is membrane protein stabilization^36,39–42^. Figure 5 shows that stabilization does indeed occur following zSMALP formation (see Supplementary Fig. 9 for a representative experiment). After a heat shock (15 min at 65 °C) essentially no MsbA in detergent remained in solution (7 ± 2%; P < 0.001 *vs* copolymers). In contrast, the protein that remained in solution after the heat shock averaged ~45 and ~70% for MsbA in 1:1 and 2:1 zSMALPs, respectively (P < 0.002 for 1:1 zSMA *vs* 2:1 zSMA). These results are not particularly surprising in that they confirmed the general finding that membrane proteins reconstituted in lipid bilayers are more stable than those in detergent micelles. However, the improved thermal stabilization in 2:1 zSMALPs *vs* 1:1 zSMALPs was somewhat surprising. It can result from a stronger interaction of the more hydrophobic 2:1 zSMA copolymer with the lipid bilayer, but understanding the bases for membrane protein stabilization will need additional studies.

**Figure 6:**
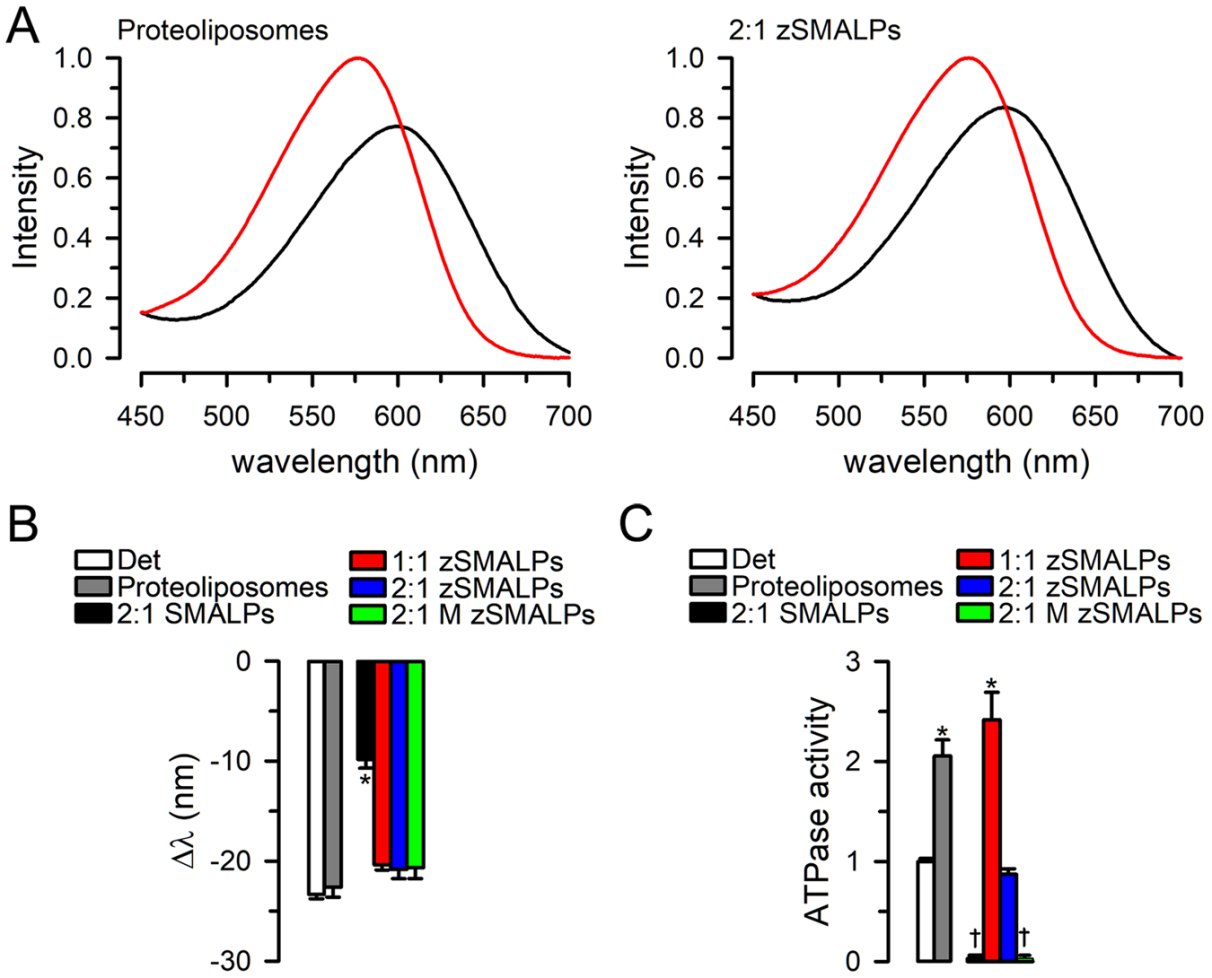
Activity of HR and MsbA reconstituted in polymer-encased nanodiscs. (**A**) Cl^−^-induced HR spectral shift. Spectral shifts were elicited by increasing Cl^−^ concentration from zero (black trace) to 250 mM by addition of NaCl (red trace). Examples of HR in proteoliposomes and 2:1 zSMALPs are shown. The intensity was normalized to the corresponding maximal intensity in 250 mM NaCl. The zSMALPs were prepared using 2:1 zSMA derived from RAFT P(St-*ran*-MA) copolymers with molecular weight of 6.4 kDa. See Supplementary Information for examples in detergent, 2:1 SMALPs, 1:1 zSMALPs and 2:1 M zSMALPs. (**B**) Cl^−^-induced HR spectral shifts summary. For each experiment, the wavelength at the maximal intensity was recorded in Cl^−^-free solution and after NaCl addition, and the differences are presented as means ± SEM (n = 3–4 *per* condition). The SMALPs, zSMALPs and M zSMALPs are the same as those described in Fig. 4. *Denotes P < 0.001 *vs* all other conditions. (**C**) MsbA ATPase activity. The ATPase activity of purified MsbA in 100 mM NaCl and 20 mM Tris/HCl, with 15% glycerol and 0.2 mM TCEP, pH 7.5, and 0.065% DDM and 0.04% sodium cholate (Det), or after reconstitution in liposomes (Proteoliposomes) or copolymer nanodiscs formed by 2:1 SMA (2:1 SMALPs), 1:1 zSMA (1:1 zSMALPs), 2:1 zSMA (2:1 zSMALPs), or 2:1 M zSMA (2:1 M zSMALPs). The SMALPs, zSMALPs and M zSMALPs are the same as those described in Fig. 4. Data are means ± SEM (n = 8–10 *per* condition), and were normalized to the average in Det (2.3 ± 0.1 ATP/s). *Denotes P < 0.001 *vs* Det; the activity in 2:1 SMALPs and 2:1 M zSMALPs was not different from zero.

We have previously demonstrated that, in contrast to membrane proteins in SMALPs, proteins reconstituted in zSMALPs can be studied at low pH (proteorhodopsin, a H^+^ pump) and in the presence of divalent cations (MsbA)^19^. Here, we compared the activity of HR and MsbA reconstituted in 1:1 and 2:1 zSMALPs. For these studies, purified HR or MsbA were first reconstituted in liposomes and then solubilized with zSMAs. The HR experienced the typical spectral blue shift between the Cl^−^-free and Cl^−^-bound states (Fig. 6A and Supplementary Fig. 10)^43,44^. The shift in wavelength maxima between the Cl^−^-free and Cl^−^-bound states was similar for HR in detergent, proteoliposomes, zSMALPs and 2:1 M zSMALP (20 ± 1 nm; n = 18; Fig. 6B). Unexpectedly the change was still present but was smaller for HR in 2:1 SMALPs (10 ± 1 nm; P < 0.001; n = 3). We presently do not have an explanation for the detrimental effect of reconstituting HR in SMALPs while HR function is well maintained in detergent, liposomes or zSMALPs. In contrast to the observations with HR, there were noticeable differences between MsbA in 1:1 and 2:1 zSMALPs. Consistent with our previous observations, the ATPase activity of MsbA (measured in 5 mM MgATP) was higher in 1:1 zSMALPs than in detergent (P < 0.001), and the protein has no activity in 2:1 SMALPs (Fig. 6C) because Mg^2+^ is required for MsbA ATPase activity and this cation causes aggregation/precipitation of SMALPs^19^. Figure 6C also shows that MsbA activity in 1:1 zSMALPs was similar to that of MsbA in liposomes. The ATPase activity data in proteoliposomes and nanodiscs are comparable since we have previously shown that MsbA is reconstituted inside-out in proteoliposomes, with the ATP-binding sites accessible to the bulk solution^6,45^. Unexpectedly, although MsbA in 2:1 zSMALPs was active, its ATPase activity was similar to that in detergent, and significantly lower than that of MsbA in 1:1 zSMALPs or proteoliposomes (P < 0.001). Even more surprising was the absence of ATPase activity in 2:1 M zSMALPs. Although we do not have a definitive explanation, it may be related to the small size of the M zSMALPs with the resulting restriction in MsbA conformational changes (Fig. 4C). The inverse relationship between MsbA thermal stability and function (Figs. 5 and 6C) in 1:1 and 2:1 zSMALPs points to a delicate balance between the copolymers structure and membrane protein stability and function that may shift depending on the different interactions among the membrane protein, lipids, and copolymers when the nanodiscs are encased within the copolymers. Although protein-polymer charge interactions are important in the functional reconstitution of membrane proteins^46^, that is not a likely factor for the difference in ATPase activity between MsbA reconstituted in 1:1 and 2:1 zSMAs. The molecular bases for the difference are unclear, but the results suggest that, at least for some membrane proteins, 1:1 zSMAs may be a better choice for functional studies even when the yield and stability of zSMALPs for 2:1 zSMAs are higher. Therefore, it will be advisable to test both copolymers since 2:1 zSMA will be preferable when increased stability is critical, whereas there may be differences in activity for a given membrane protein depending on the St:MAs. Understanding the effect on stability and function by different copolymers will need additional research. Such studies will be important to rationally design new and improved copolymers for specific applications.

## Conclusions

SMALPs have gained popularity as membrane protein platforms because SMA copolymers can solubilize membranes directly, bypassing the use of detergent, and produce nanodiscs where the membrane patch includes the native lipids^14–16^. SMAs can also be used on purified membrane proteins reconstituted in liposomes of predetermined composition for studies of the effects of lipids on membrane protein structure and function^14–16^. Buffer incompatibilities of SMAs and SMALPs constitute a major problem when polyvalent cations at mM concentration or low pH are needed^14,17–19^. Newer copolymers that offer distinct advantages over the traditional SMA copolymers have been studied recently^19,47–50^. Diisobutylene/maleic acid copolymer (DIBMA), poly(styrene-co-maleimide) (SMI), styrene maleimide quaternary ammonium (SMA-QA) and our zSMAs are compatible with high concentrations of divalent cations^19,47–49^. SMI can solubilize membrane proteins into nanoparticles efficiently at pH < 7.5, but not at more alkaline pHs, and the smaller diameter of the “SMILPS” could limit its application to “small” proteins^48^. In contrast, our zSMAs are stable at acidic and alkaline pH, and zSMAs with molecular weights ranging from ~10,000 to 45,000 can produce zSMALPs of increasing sizes^19^.

Here, we showed the formation of polymer-encased nanodiscs by new zSMA and M zSMA copolymers under a variety of conditions. We found significant effects on solubilization by temperature, incubation time, polymer concentration, polymer chain size and microstructure, salt concentration, and pH. We also found that the membrane and membrane protein are additional players, but systematic studies will be needed to define their precise roles. Based on the variables explored, we suggest the use of 1% copolymer, intermediate ionic strength (150–500 mM NaCl), pH in the 7.5–8.3 range, incubation at RT or above, and an incubation time of ~2 h. The differences among different copolymers appeared to depend on the copolymer structure, membrane structure/composition, and/or membrane protein. In general, zSMAs performed better than M zSMAs under similar conditions, with remarkable differences in MsbA activity between the zSMALPs encased by the two different families of copolymers, suggesting an important role of the polymer chain size distribution and microstructure in nanodisc formation and support of membrane protein function. Among all the zwitterionic copolymers, those with a 2:1 St:MA ratio showed better solubilization efficiency than those with a 1:1 St:MA ratio, and a higher stability of the corresponding zSMALPs, but improved efficiency and stability do not translate into a higher membrane protein activity, as clearly showcased in the example of MsbA. As for the polymer chain sizes, the results showed that P(St-*ran*-MA) copolymers in the 3- to 12-kDa size range can be safely used as a starting point for the preparation of nanodiscs.

The overall conclusion is that solubilization by the new 1:1 and 2:1 zSMA, and 1:1, 2:1 and 3:1 M zSMAs to a large extent, is quite forgiving and better than that by 2:1 SMA, a benchmark for the SMALP studies due to the recognition that the solubilization efficiency by the 2:1 SMAs is better than that of the 1:1 SMAs^23,27,29^. This is important because, in principle, solubilization conditions already established for a membrane protein using SMAs or detergents can be used with few or no changes to solubilize them into zSMALPs with reasonable results, minimizing the need for extensive trials. In summary, the new zwitterionic copolymers developed and presented here provide new tools for membrane-protein studies in polymer-encased nanodiscs, and solid bases for further development of improved zSMA copolymers.

## Materials and Methods

### Materials

Styrene (St, inhibited with ~0.005% 4-tert-butylcatechol, Sigma-Aldrich) was purified by passing it through a basic aluminum oxide column before use. Maleic anhydride (MA, puriss p.a., ACS reagent, ≥99.0%, Sigma-Aldrich) was used as received. Azobisisobutyronitrile (AIBN, 98%, Sigma-Aldrich) was re-crystallized from ethanol twice before use. *S*-1-dodecyl-S′-(α,α′-dimethyl-α″-acetic acid) trithiocarbonate (DATC) was synthesized according to literature^51^. The commercially available SMA precursors (Lipodisq copolymers with 1:1, 2:1 and 3:1 St:MA) were gifts from Malvern Cosmeceutics (WR14 3SZ, United Kingdom). All other chemicals were reagent grade purchased from Sigma-Aldrich and used as received.

### Synthesis of zSMA *via* reversible addition−fragmentation chain-transfer (RAFT) polymerization

The reaction scheme used to prepare zSMAs is shown in Fig. 1A. We first prepared the random copolymers of styrene-maleic anhydride, P(St-*ran*-MA), with different St:MA ratios by controlling the stoichiometry of the two monomers^19^. In a typical reaction to prepare 2:1 P(St-*ran*-MA) with a molecular weight of ~6 kDa, styrene (2.08 g, 20 mmol), maleic anhydride (0.98 g, 10 mmol), DATC (36.4 mg, 0.10 mmol) and AIBN (3.3 mg, 0.02 mmol) were dissolved in 3.0 g THF in a 10-mL Schlenk flask, stirred, and degassed by three freeze-pump-thaw cycles. The flask was sealed and immersed in a 65 °C-oil bath, and after a predetermined time of ~50 min the reaction was quenched by liquid nitrogen, such that the conversion of styrene was controlled to ~20%. The reaction time was determined by the kinetics study commonly used in controlled/“living” polymerization^52^. The mixture was then diluted with THF and precipitated three times in a mixture of ether/chloroform (3/1 v/v). The molecular weights and polydispersity indices of different zSMA copolymers are summarized in Table 1.

To synthesize cysteamine modified phosphatidylcholine, 2-methacryloyloxyethyl phosphorylcholine (PC) was reacted with cysteamine *via* a thiol-ene “click” reaction (Fig. 1A). Briefly, cysteamine (0.52 g, 6.77 mmol), 2-methacryloyloxyethyl phosphorylcholine (2.0 g, 6.77 mmol) and dimethylphenylphosphine (46.9 mg, 0.34 mmol) were dissolved in 12 mL of dimethylsulfoxide (DMSO) in a 25-mL flask. After stirring for 2 days at RT the solution was precipitated twice in cold acetone/ether (2/1 v/v). The product was collected by centrifugation and dried in vacuum overnight.

Finally, P(St-*ran*-MA) was modified with the cysteamine-PC to prepare the zSMAs. In a typical run, cysteamine-PC (0.36 g, 0.97 mmol) and dicyclohexylcarbodiimide (DCC) (0.20 g, 0.97 mmol) were dissolved in 10 mL of DMSO. SMA (0.093 g, 0.32 mmol maleic anhydride) dissolved in 10 mL of DMSO was added dropwise into the mixture. After stirring for 2 days at RT the mixture was dialyzed against Millipore water. After two days, the undissolved white solid was filtered away and the solution was dried in a lyophilizer.

### Synthesis of zSMA using the Lipodisq P[St-*ran*-MA] copolymers from Malvern Cosmeceutics

We adopted a similar approach to prepare M zSMAs, as shown in Fig. 1B, except that the P(St-*ran*-MA) copolymers with 1:1, 2:1 and 3:1 St:MA were obtained from Malvern Cosmeceutics. The molecular weights and polydispersity indices of the different Lipodisq P(St-*ran*-MA) copolymers are summarized in Table 1. As controls, we also prepared SMAs (Malvern) using the same Lipodisq P[St-*ran*-MA] copolymers as precursors.

### Polymer characterizations

Gel permeation chromatography (GPC) analysis was performed on an Agilent 1260 HPLC system equipped with a diode array UV detector, a Wyatt Optilab REX refractive index detector, and a Wyatt miniDAWN TREOS multiangle light scattering detector. An Agilent PLgel 5 μm MIXED-D column was used with dimethylformamide (DMF) with 0.01 M ammonium acetate as eluent for SMAs and THF for PSt, at a flow rate of 0.5 mL/min. ^1^H NMR was performed on a 400-MHz JEOL liquid-state NMR spectrometer.

### Expression, purification and reconstitution of halorhodopsin

A synthetic gene (Genscript, Piscataway, NJ) coding for *Natronomonas pharaonis* halorhodopsin (HR) fused at the C-terminal end to a 6-His tag followed by a Flag tag (Supplementary Fig. 11) was cloned into the *NcoI*/*BamHI* sites of the expression vector pET19b (Novagen, Madison, WI). HR was overexpressed in the *E. coli* strain BL21 (DE3) (Agilent Technologies, Santa Clara, CA) transformed with the pET19-HR plasmid. The cells were grown at 37 °C in 2YT medium with 200 µg/mL ampicillin, and 10 µM all-*trans* retinal was added at the time of induction (at OD_600_ ~1) with 1 mM isopropyl-β-D-thiogalactopyranoside (IPTG). The cells were harvested 4 h later, and all subsequent procedures were performed at 4 °C unless specified otherwise. Cell pellets were resuspended in 50 mM Tris/HCl and 5 mM MgCl_2_, pH 8, with 10 µg/mL lysozyme, 10 µg/mL DNAse I, 1 mM phenylmethanesulfonyl fluoride (PMSF) and a protease inhibitor cocktail (1 tablet/100 mL of buffer; complete EDTA-free, Roche), and lysed on a microfluidizer. Crude membranes were prepared by centrifugation at 135,000 g for 1.5 h, and were solubilized for 2 h at 4 °C in a buffer containing 300 mM NaCl, 50 mM MES (2-(*N*-morpholino)ethanesulfonic acid), 5 mM imidazole, 1 mM PMSF and 1.5% DDM (Inalco Pharmaceuticals, San Luis Obispo, CA), pH 6.5, at a total protein concentration <3 mg/mL. The DDM-solubilized lysate was centrifuged at 100,000 g for 30 min, and the supernatant was incubated overnight with Talon Co^2+^ beads (Talon Superflow, Clontech, Mountain View, CA). The resin was washed first with 10 column volumes of a buffer containing 300 mM NaCl, 50 mM MES, pH 6.5, 0.1% DDM and 45 mM imidazole, and then with 10 column volumes of the same buffer, but with NaCl increased to 1 M. The last wash was with 4 column volumes of 300 mM NaCl, 50 mM MES, 0.1% DDM and 65 mM imidazole, pH 6.5. Imidazole was added to a concentration of 200 mM for elution. After elution, the imidazole was removed by exchanging the buffer to 50 mM sodium phosphate and 50 mM sodium citrate, with 0.1% DDM, pH 7. The purity of the preparation was assessed by staining gels (16% SDS PAGE) with Instant Blue (Expedeon, San Diego, CA) and by UV-Vis spectroscopy. The HR samples were concentrated to ~1 mg/mL and were stored at −80 °C until use. For reconstitution into liposomes, purified HR solubilized in DDM was mixed with *E. coli* polar lipids (Avanti Polar Lipids, Alabaster, AL) at a 1:3 protein:lipid ratio (w/w), and proteoliposomes were formed by detergent removal by gel filtration on columns (Zeba columns, Thermo Fisher Scientific, Waltham, MA) pre-equilibrated with 200 mM NaSO_4_ and 50 mM sodium phosphate, pH 7. After reconstitution, the samples were extruded through a 200-nm polycarbonate filter (Mini-Extruder, Avanti Polar Lipids).

### Expression, purification and reconstitution of MsbA

A fully-active MsbA mutant (Supplementary Fig. 11) was expressed and purified as previously described^6,45^. Briefly, MsbA expression in BL21 DE3-RILP *E. coli* cells (Agilent Technologies) was induced for 4 h at 30 °C with 1 mM IPTG at an OD_600_ of ~1. Crude membranes were prepared as described above for HR, and were solubilized for 1 h at RT with 2% DDM and 0.04% sodium cholate in a buffer containing 100 mM NaCl, 20 mM Tris/HCl, with 15% glycerol, 0.5 mM Tris (2-carboxyethyl) phosphine hydrochloride (TCEP) and 1 mM PMSF, pH 8. Solubilized MsbA was purified by metal affinity chromatography (Talon Superflow; Clontech) followed by size-exclusion chromatography using a Bio-Scale Mini Bio-Gel P-6 Desalting Cartridge (Bio-Rad, Hercules, CA) equilibrated with storage buffer: 100 mM NaCl, 20 mM Tris/HCl, 0.065% DDM, 0.04% sodium cholate, with 15% glycerol and 0.2 mM TCEP, pH 7.5. MsbA was stored at −80 °C until use. Protein concentration was determined by absorbance at 280 nm and purity was estimated at >95% from SDS-PAGE gels stained with Instant Blue (Expedeon). Reconstitution of purified MsbA into liposomes formed by *E. coli* polar lipids was performed by gel filtration and extrusion, as described for HR, but using a 1:10 protein:lipid ratio (w/w) in 100 mM NaCl, 20 mM Tris/HCl, with 0.1 mM TCEP, pH 7.4.

### Extraction of HR and MsbA from crude membranes and proteoliposomes with copolymers

For the basic protocol, crude membranes from *E. coli* expressing HR or MsbA were resuspended at a final concentration of 30 mg/mL (wet membrane mass; total protein <3 mg/mL) in 500 mM NaCl and 50 mM Tris/HCl, with 10% glycerol, pH 7.5. Copolymers were added to a final concentration of 1% (w/v) and the samples were incubated with gentle rotation for 2 h at RT for HR or at 37 °C for MsbA. Insoluble material was removed by centrifugation at 100,000 g for 45 min at 4 °C, and the supernatant containing HR- or MsbA-loaded zSMALPs was analyzed by Western blotting using an antibody against the His tag that is conjugated to Alexa Fluor 647 (HisTag Antibody [iFluor 647], GenScript). The signal was visualized using an Odyssey Infrared Imager (Li-Cor Biosciences). The efficiency of protein solubilization was quantified from the Western blots by densitometry (UN-SCAN-IT, Silk Scientific). In some experiments, copolymer concentration was increased to 2.5 or 5%, NaCl concentration was decreased to 150 mM, or pH was changed to 6.5 or 8.3, as indicated in the text. For extraction by copolymers of HR or MsbA reconstituted into liposomes we used the conditions of the corresponding basic protocols described above.

### HR and MsbA activity assessment

We used the blue shift in absorption spectra elicited by NaCl as an indicator of HR response to Cl^−^ binding^43,44^. Absorption spectra of HR in DDM, liposomes or polymer-encased nanodiscs were collected on a spectrophotometer (Jasco V-630, Easton, MD) at 22 °C in absence of Cl^−^ and after addition of 250 mM NaCl. The ATPase activity of MsbA was measured using a variant of the ATPase linked assay^45,53^. In this system, regeneration of the ATP consumed by MsbA is tied to the conversion of NADH to NAD^+^, and hydrolysis activity is determined by the decline in NADH measured by the absorbance at 340 nm. Measurements were performed at 37 °C under conditions approaching V_max_, using 0.5 to 1 µg of MsbA in 200 μL of linked enzyme cocktail containing: 100 mM NaCl, 20 mM Tris/HCl pH 7.5, 12 mM MgSO_4_, 5 mM ATP, 0.1 mM EGTA, 3 mM phosphoenolpyruvate, 0.1 mM TCEP, 25 μg/mL lactate dehydrogenase, 50 μg/mL pyruvate kinase and ~0.7 mM NADH. For measurements in detergent the reaction also contained 0.065% DDM and 0.04% sodium cholate. For the measurements, MsbA-loaded nanodiscs were enriched based on the affinity of the His-tagged MsbA for Ni^2+^. The supernatant from the solubilization of proteoliposomes was mixed with Ni-NTA beads (Thermo Fisher Scientific) at a ratio of 100 µL resin/mL solubilized protein, and after incubation at 4 °C overnight with gentle rotation the samples were transferred to a gravity flow column. The resin was washed with 10 column volumes of 100 mM NaCl and 20 mM Tris/HCl, with 0.1 mM TCEP and 20 mM imidazole, pH 7.4, and elution was achieved by increasing imidazole to 200 mM. Eluted fractions were analyzed on gels (16% SDS-PAGE) stained with Instant Blue (Expedeon) and used for the ATPase measurements.

### Estimation of nanodiscs size by dynamic light scattering (DLS)

Measurements were performed at 22 °C on a Zetasizer Nano ZSP (Malvern Instruments, Westborough, MA) using 40-µL disposable microcuvettes. Size-number distributions were generated using the Zetasizer software version 7.11 and were analyzed using the protein analysis distribution.

### Statistics

Statistical comparisons were performed by the Student’s *t* test for paired or unpaired data, or one-way analysis of variance, as appropriate. P < 0.05 in a two-tailed analysis was considered significant. The number of experiments given in the main text and figure legends corresponds to independent measurements.

## Supplementary information

Supplementary Information.

## Data Availability

The datasets generated during and/or analyzed during the current study are available from the corresponding authors on reasonable request.
