# Supplementary material for: Extraction and reconstitution of membrane proteins into lipid nanodiscs encased by zwitterionic styrene-maleic amide copolymers
Source: Sci Rep. 2020 Jun 18;10:9940. doi: 10.1038/s41598-020-66852-7 (PMC7303149; doi:10.1038/s41598-020-66852-7)
Supplement: Supplementary file 1 — Supplementary Information. [file 41598_2020_66852_MOESM1_ESM.pdf]

SUPPLEMENTARY INFORMATION

Extraction and reconstitution of membrane proteins into lipid nanodiscs encased by zwitterionic styrene-maleic amide copolymers

Mariana C Fiori, Wan Zheng, Elizabeth Kamilar, Geuel Simiyu, Guillermo A Altenberg & Hongjun Liang

Department of Cell Physiology and Molecular Biophysics, and Center for Membrane Protein Research, Texas Tech University Health Sciences Center, Lubbock, Texas, USA.

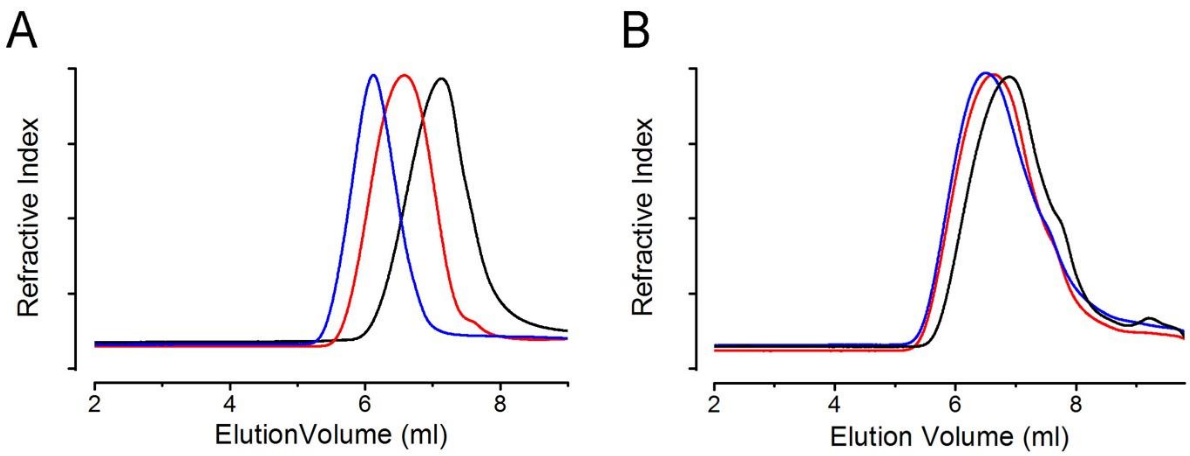

Supplementary Figure 1. Gel permeation chromatography (GPC) of P(St-*ran*-MA) copolymers. (A) P(St-*ran*-MA) copolymers prepared by RAFT polymerization (St:MA ~2:1) with different molecular weights and polydispersity indices: $\overline{M_n}$(kDa)/*PDI* = 3.1/1.05 (black), 6.4/1.04 (red), and 12.0/1.13 (blue), respectively. (B) Lipodisq P(St-*ran*-MA) copolymers with different St:MA ratios, molecular weights and polydispersity indices. Black: St:MA ~1:1, $\overline{M_n}$ (kDa)/*PDI* = 4.6/1.22; red: St:MA ~2:1, $\overline{M_n}$ (kDa)/*PDI* = 5.0/1.52; blue: St:MA ~3:1, $\overline{M_n}$(kDa)/*PDI* = 5.7/1.59.

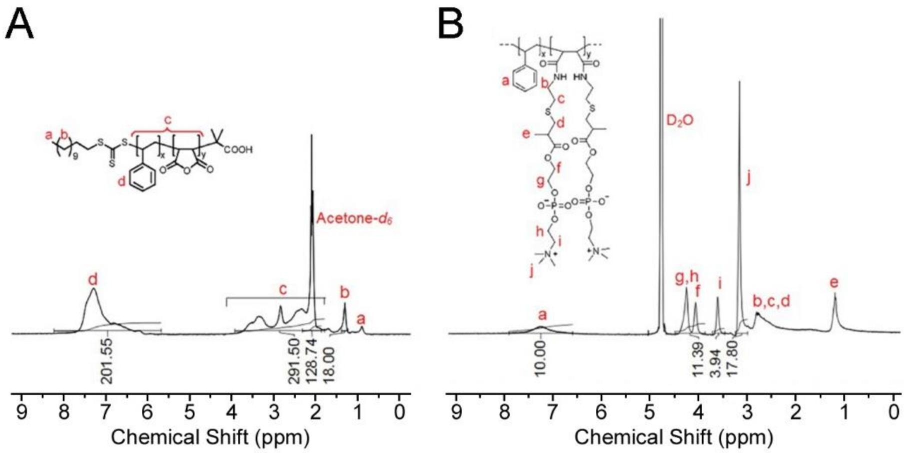

Supplementary Figure 2. Structural characterization of zSMA. Comparison of the ^{1}H NMR spectra of P(St-*ran*-MA) prepared by RAFT polymerization (St:MA=2:1, $\overline{M_n}$ = 6.4 kDa) before (A; acetone-*d*$_6$ was used as solvent) and after its conversion to zSMA (B; D_2O was used as solvent). In panel A, the peak *b (18 H)* was assigned to alkyl protons on the DATC moiety, and peaks *c* within the δ =1.78~4.0 ppm range were attributed to alkyl protons of the St and MA repeating units. Peak *d* was assigned to the benzyl protons, from which an average styrene-repeating-unit number of 40 was obtained. After subtracting the peak of acetone-*d*$_6$ from peaks *c*, the St:MA ratio was calculated as 1.93:1.00 according to:

$$\frac{3x+2y}{5x} = \frac{291.50-128.74}{201.55} \qquad (1)$$

where *x* and *y* are the number of St and MA repeating units, respectively. Based on the NMR analysis, we identified this copolymer as P(St$_{40}$-*ran*-MA$_{21}$); Panel B is the ^{1}H NMR spectrum of the zSMA derived from cysteamine-PC modified P(St$_{40}$-*ran*-MA$_{21}$). The styrene structure was not affected by the modification and was used as reference to calculate the percentage of amide bond formation on individual maleic anhydride repeating units due to their reactions with cysteamine-PC. The number of benzyl protons located at 6.60~7.90 ppm (peak *a*) from the styrene structure were set to 10. Since St:MA=1.93 (see panel A), reaction of both carboxyl moieties on each maleic anhydride unit with cysteamine-PC should yield a total of 12.43 protons for peaks *f*, *h* and *g*. Given that a total of 11.39 protons were obtained from the integration of peaks *f*, *h*, and *g*, the percentage of amide bond formation was calculated as ~92%. Alternatively, if we used peak *i* for this estimation, which is expected to have 4.15 protons, the percentage of amide bond formation was calculated as ~95%. From these data we concluded that the cysteamine-PC modification of P(St-*ran*-MA) copolymers to convert them into zSMA was highly effective. The slight deviation from 100% amide bond formation could be due to a small percentage of unreacted carboxy acid moiety and/or imide formation during the cysteamine-PC modification. Nevertheless, this deviation is insignificant. See **Table 1** for details on the copolymers.

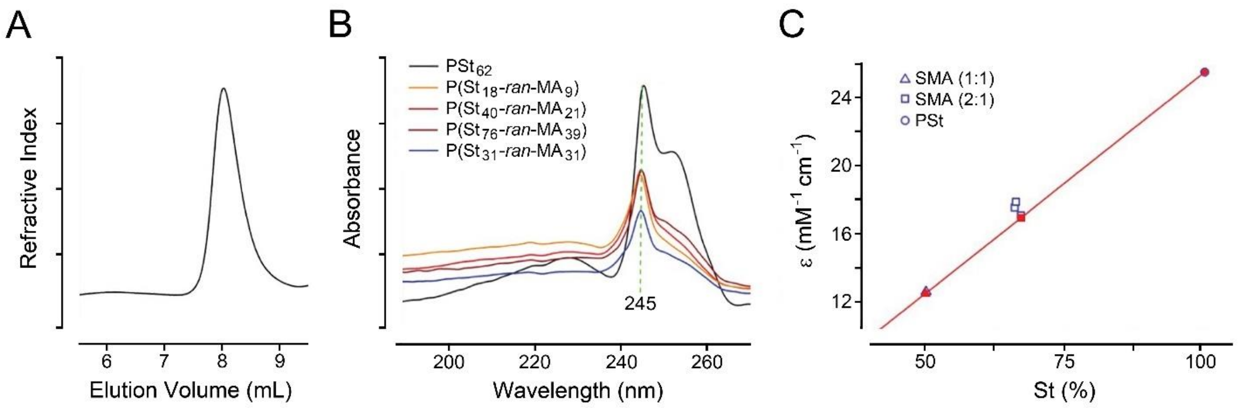

Supplementary Figure 3. Characterization of the St:MA ratios by UV spectroscopy. (A) GPC of the well-defined polystyrene PSt_{62} prepared by RAFT polymerization (M_n = 6,770 Da, PDI = 1.06). (B) UV spectra of well-defined SMAs and PSt_{62} prepared by RAFT polymerization. The different St:MA ratios of SMAs were identified by ^1H NMR analysis, and each spectrum was normalized by the molar concentration of the respective polymer in THF. The characteristic peak at 245 nm (marked by dotted line) is from the St moiety in the polymers. (C) Relationship between the molar absorption coefficient of PSt_{62} at 245 nm (ε_{245}) and the molar fraction of the St moiety in the polymers (St (%)). Using the ε_{245} of PSt_{62} as reference, we calculated the ε_{245} of SMAs as St% in the polymers (red line) and compared that with the measured ε_{245} of SMAs with different St:MA ratios. The triangle, square, and circle represent a St% of 50% (St:MA ~1:1), 67% (St:MA ~2:1), and 100% (PSt), respectively. The calculated values expected from ^1H NMR analysis at these St% are shown as solid red symbols and the experimental values as empty blue symbols. The agreement between the expected and experimental ε_{245} values supports the St% determined by ^1H NMR analysis.

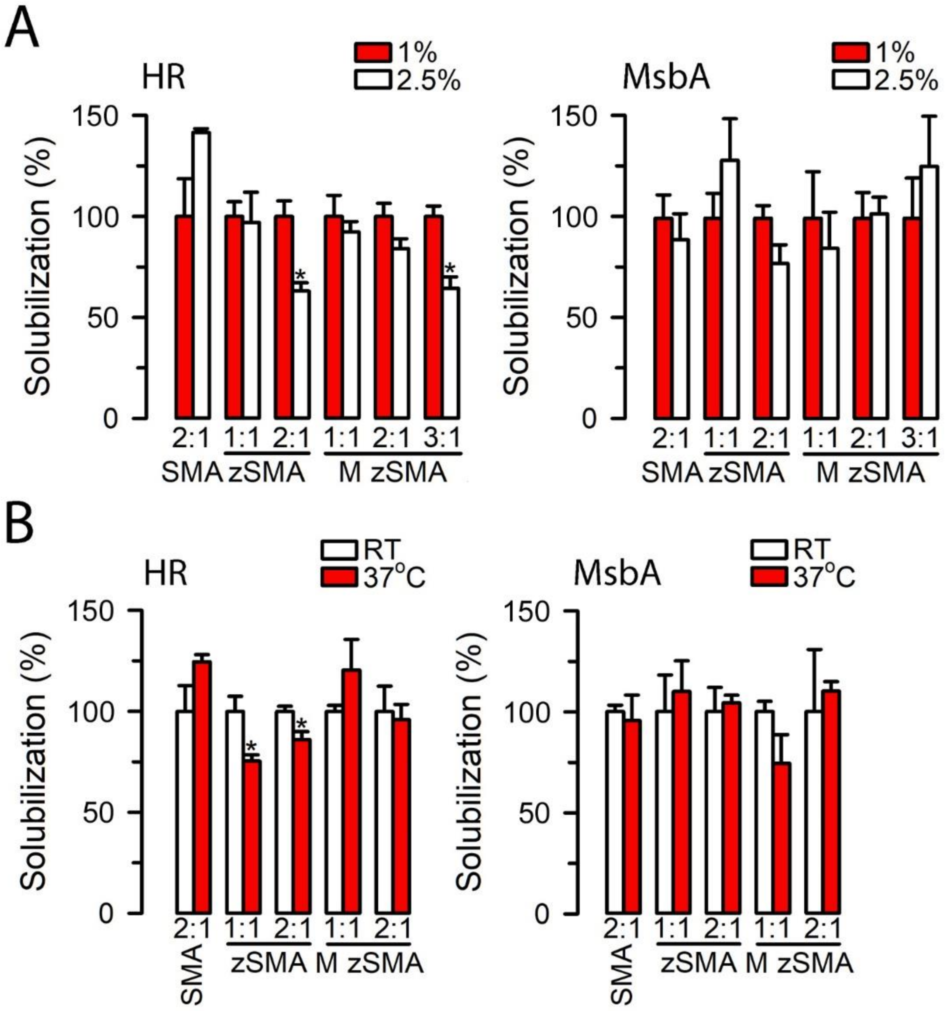

Supplementary Figure 4. Effects of copolymer concentration and temperature on HR and MsbA solubilization. The basic solubilization protocol consisted of incubation in 500 mM NaCl and 50 mM Tris/HCl, with 10% glycerol, pH 7.5, and a final copolymer concentration of 1% (w/v). Solubilization of the crude membranes proceeded for 2 h at RT for HR and at 37ºC for MsbA. After the incubation the samples were centrifuged at 100,000 g for 45 min, and the % of solubilized HR was calculated from densitometry analysis of Western blots (anti-His antibody). (A) Effects of copolymer concentration. Solubilization conditions were those described above, except that copolymer concentrations were 1 or 2.5% (see text for 5%). The 2:1 SMA was derived from Malvern P(St-*ran*-MA) with molecular weight of 5.0 kDa, while the 1:1 and 2:1 zSMA were derived from RAFT P(St-*ran*-MA) with molecular weights of 6.7 and 6.4 kDa, respectively. The M zSMA samples are as described in **Table 1**. Data were normalized to the corresponding 1% average value. * denotes $P<0.01$ *vs* the corresponding 1% values. (B) Effects of incubation temperature. Solubilization conditions were those of the basic protocol, except that incubations were performed at RT or 37ºC. Data were normalized to the corresponding RT average value. * denotes $P<0.05$ *vs* the corresponding RT value. For all panels, data are means ± SEM (n = 3-5 *per* condition).

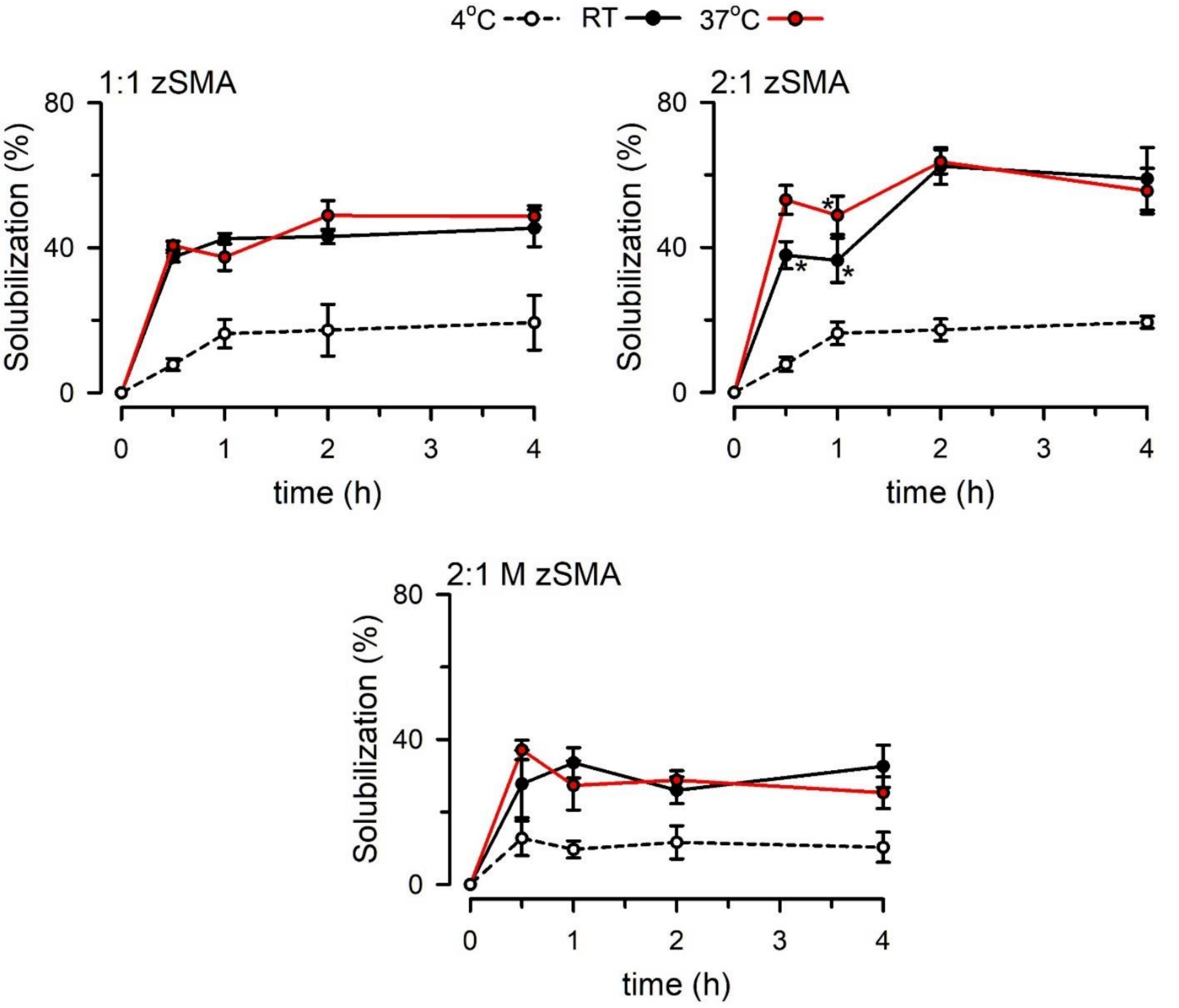

Supplementary Figure 5. Dependence of MsbA solubilization on temperature and time. Solubilization of MsbA from crude membranes was performed in 500 mM NaCl and 50 mM Tris/HCl, with 10% glycerol, pH 7.5, and a final copolymer concentration of 1% (w/v). After incubation the samples were centrifuged at 100,000 g for 45 min, and the % of solubilized MsbA was calculated from densitometry analysis of Western blots using an anti-His antibody. * denotes $P<0.03$ *vs* the 2-h value at the corresponding temperature. Data are means ± SEM; 1:1 zSMA: n = 3-7; 2:1 zSMA: n = 4-9; 2:1 M zSMA: n = 3-4.

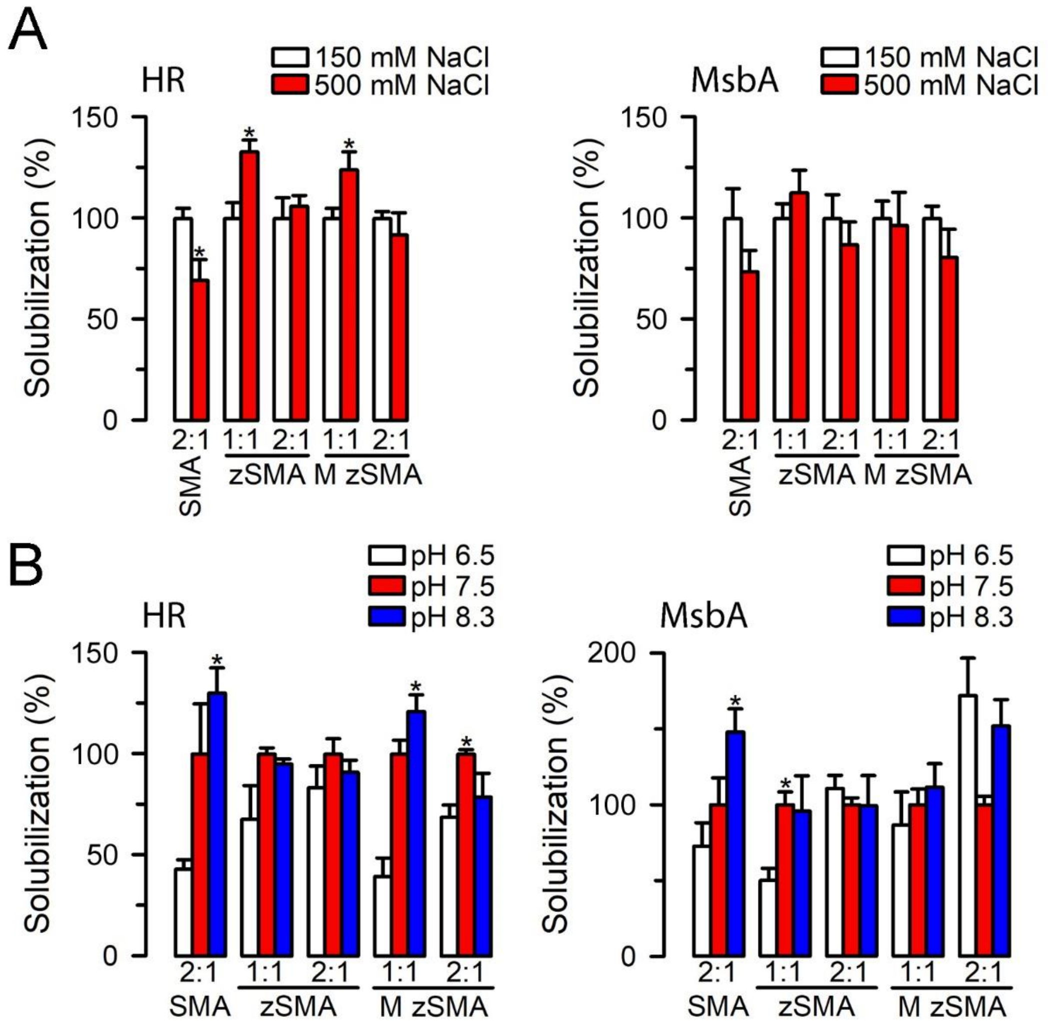

Supplementary Figure 6. Effects of salt concentration and solution pH on HR and MsbA solubilization. (A) Effects of [NaCl]. Solubilization conditions were those of the basic protocol, except that [NaCl] was 150 or 500 mM. Data were normalized to the corresponding 150 mM average value. The 2:1 SMA was derived from Malvern P(St-*ran*-MA) with molecular weight of 5.0 kDa, while the 1:1 and 2:1 zSMA were derived from RAFT P(St-*ran*-MA) with molecular weights of 6.7 and 6.4 kDa, respectively. The M zSMA samples are as described in **Table 1**. * denotes significant difference *vs* the corresponding 150 mM value (SMA and M zSMA $P<0.05$; 1:1 zSMA $P<0.01$). (B) Effects of solution pH. Solubilization conditions were those described above, except that the pH of the solubilization solutions was 6.5, 7.5 or 8.3. Data were normalized to the corresponding pH 7.5 average value. * denotes significant difference *vs* the corresponding pH 6.5 value; HR: $P<0.01$ for SMA, 1:1 M zSMA and 2:1 M zSMA $P<0.01$; MsbA: $P<0.05$ for zSMA, and $P<0.01$ for 1:1 zSMA. For all panels, data are means ± SEM (n = 3-6 *per* condition). See **Supplementary Figure 4** for details.

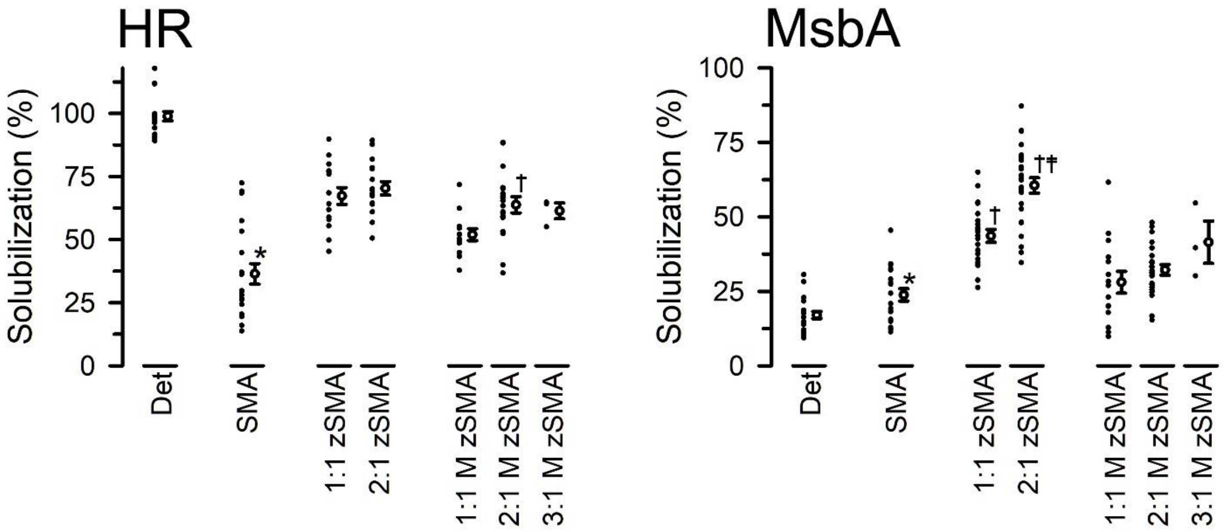

Supplementary Figure 7. Solubilization of recombinant HR and MsbA from *E. coli* crude membranes. Individual solubilization experiments performed over the course of ~2 years. See **Fig. 2** for details. The symbols with the errors correspond to the means ± SEM and the small symbols to the individual experiments.

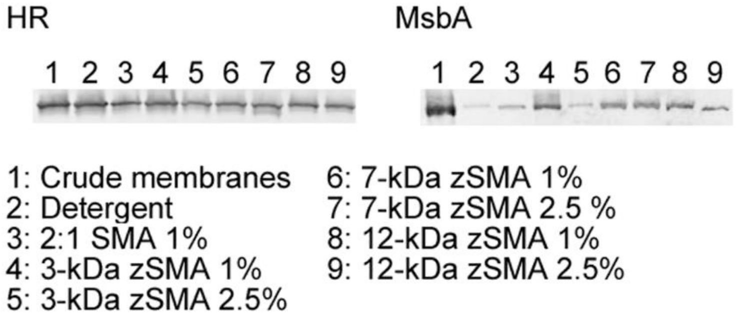

Supplementary Figure 8. Effects of 2:1 zSMA copolymers of different molecular weights on solubilization of HR (left) and MsbA (right) into zSMALPs. Bands in all lanes correspond to the same volume. After solubilization of the crude membranes the supernatant was collected and run to determine the percentage of solubilized HR or MsbA. The concentrations tested for each copolymer were 1 and 2.5% (w/v) as indicated in the figure. The data correspond to representative Western blots using an anti-His tag antibody. See Materials and Methods for details. Densitometry analysis of blots such as this yielded the data in **Fig. 3** of the manuscript.

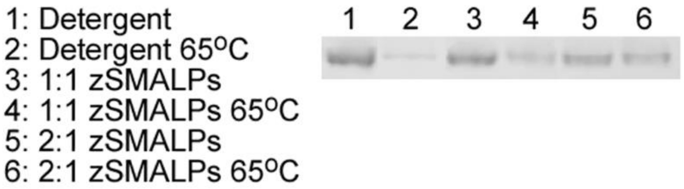

Supplementary Figure 9. Thermal stability of solubilized MsbA. Representative Western blot (anti-His tag antibody) showing MsbA in solution after a 15-min 65ºC heat shock. MsbA was studied in detergent and after reconstitution into 1:1 and 2:1 zSMALPs. Densitometry analysis of blots such as this yielded the data in **Fig. 5** of the manuscript.

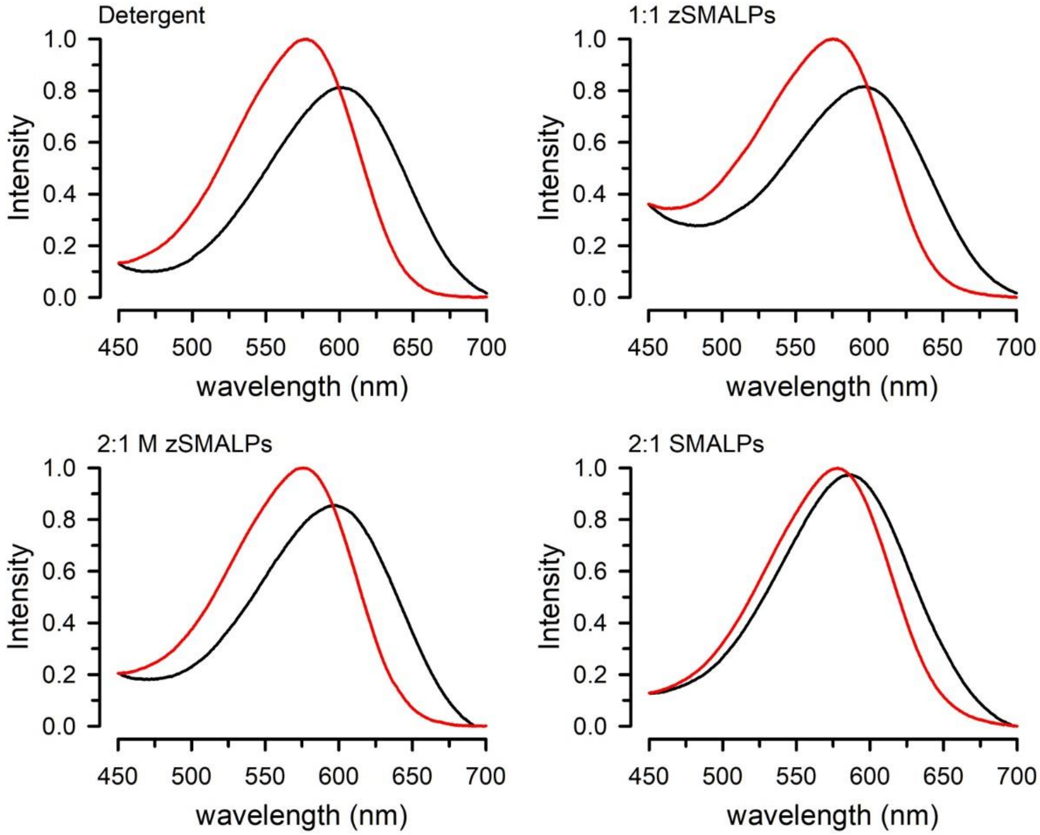

Supplementary Figure 10. Cl^--induced HR spectral shift. Spectral shifts were elicited by increasing the concentration of Cl^- from zero to 250 mM (addition of NaCl). Examples of HR in detergent (DDM), 1:1 zSMALPs, 2:1 M zSMALPs and 2:1 SMALPs are shown. The 2:1 SMA used to prepare SMALPs was derived from Malvern P(St-*ran*-MA) with molecular weight of 5.0 kDa, while the 1:1 and 2:1 zSMA used to prepare zSMALPs were derived from RAFT P(St-*ran*-MA) with molecular weights of 6.7 and 6.4 kDa, respectively. The intensity was normalized to the corresponding maximal intensity in NaCl.

Halorhodopsin (HR)

1	MAETLPPVTE	SAVALQAEVT	QRELFEFVLN	DPLLASSLYI	NIALAGLSIL
51	LFVFMTRGLD	DPRAKLIAVS	TILVPVVSIA	SYTGLASGLT	ISVLEMPAGH
101	FAEGSSVMLG	GEEVDGVVTM	WGRYLTWALS	TPMILLALGL	LAGSNATKLF
151	TAITFDIAMC	VTGLAAALTT	SSHLMRWFWY	AISCACFLVV	LYILLVEWAQ
201	DAKAAGTADM	FNTLKLLTVV	MWLGYPIVWA	LGVEGIAVLP	VGVTSWGYSF
251	LDIVAKYIFA	FLLLNYLTSN	ESVVSGSILD	VPSASGTPAD	DLEENLYFQG
301	GSDYKDDDDK	GSGHHHHHH			

MsbA

1	MGHHHHHHHH	HHSSGHIDDD	DKHMHNDKDL	STWQTFRRLW	PTIAPFKAGL
51	IVAGIALILN	AASDTFMLSL	LKPLLDDGFG	KTDRSVLLWM	PLVVIGLMIL
101	RGITSYISSY	AISWVSGKVV	MTMRRRLFGH	MMGMPVAFFD	KQSTGTLLSR
151	ITYDSEQVAS	SSSGALITVV	REGASIIGLF	IMMFYYSWDL	SIILVVLAPI
201	VSIAIRVVSK	RFRSISKNMQ	NTMGQVTTSA	EQMLKGHKEV	LIFGGQEVET
251	KRFDKVSNKM	RLQGMKMVSA	SSISDPIIQL	IASLALAFVL	YAASFPSVMD
301	SLTAGTITVV	FSSMIALMRP	LKSLTNVNAQ	FQRGMAAAQT	LFAILDSEQE
351	KDEGKRVIDR	ATGDLEFRNV	TFTYPGREVP	ALRNINLKIP	AGKTVALVGR
401	SGSGKSTIAS	LITRFYDIDE	GHILMDGHDL	REYTLASLRN	QVALVSQNVH
451	LFNDTVANNI	AYARTEEYSR	EQIEEAARMA	YAMDFINKMD	NGLDTIIGEN
501	GVLLSGGQRQ	RIAIARALLR	DSPILILDEA	TSALDTESER	AIQAALDELQ
551	KNRTSLVIAH	RLSTIEQADE	IVVVEDGIIV	ERGCHSELLA	QHGVYAQLHK
601	MQFGQ				

Supplementary Figure 11. Sequences of the halorhodopsin and MsbA used in this study. HR has C-terminal Flag and poly-His sequences preceded by a TEV protease site. MsbA has an N-terminal poly-His tag followed by an enterokinase cleavage sequence, replacement of two Cys with Ala, and introduction of a single Cys (Thr 561 to Cys). All modifications are underlined.
